# Slicing Algorithm and Partition Scanning Strategy for 3D Printing Based on GPU Parallel Computing

**DOI:** 10.3390/ma14154297

**Published:** 2021-07-31

**Authors:** Xuhui Lai, Zhengying Wei

**Affiliations:** State Key Laboratory for Manufacturing System Engineering, Xi’an Jiaotong University, Xi’an 710049, China; laikeyo@stu.xjtu.edu.cn

**Keywords:** GPU slice, MS algorithm, curve interpolation, partition, additive manufacturing, scan order

## Abstract

Aiming at the problems of over stacking, warping deformation and rapid adjustment of layer thickness in electron beam additive manufacturing, the 3D printing slicing algorithm and partition scanning strategy for numerical control systems are studied. The GPU (graphics processing unit) is used to slice the 3D model, and the STL (stereolithography) file is calculated in parallel according to the normal vector and the vertex coordinates. The voxel information of the specified layer is dynamically obtained by adjusting the projection matrix to the slice height. The MS (marching squares) algorithm is used to extract the coordinate sequence of the binary image, and the ordered contour coordinates are output. In order to avoid shaking of the electron gun when the numerical control system is forming the microsegment straight line, and reduce metal overcrowding in the continuous curve C^0^, the NURBS (non-uniform rational b-splines) basis function is used to perform curve interpolation on the contour data. Aiming at the deformation problem of large block components in the forming process, a hexagonal partition and parallel line variable angle scanning technology is adopted, and an effective temperature and deformation control strategy is formed according to the European-distance planning scan order of each partition. The results show that the NURBS segmentation fits closer to the original polysurface cut line, and the error is reduced by 34.2% compared with the STL file slice data. As the number of triangular patches increases, the algorithm exhibits higher efficiency, STL files with 1,483,132 facets can be cut into 4488 layers in 89 s. The slicing algorithm involved in this research can be used as a general data processing algorithm for additive manufacturing technology to reduce the waiting time of the contour extraction process. Combined with the partition strategy, it can provide new ideas for the dynamic adjustment of layer thickness and deformation control in the forming process of large parts.

## 1. Introduction

Electron beam additive manufacturing (EBAM) uses metal wires or strips as raw materials to rapidly form metal components with mechanical properties equivalent to castings, and is widely used in the rapid manufacturing of precision blanks for large parts, such as aerospace, automobile manufacturing, etc. [1,2,3,4,5,6,7]. However, with the increase of the area of the forming area, the temperature gradient at both ends of the scanning line increases sharply [8,9,10,11,12,13,14]. When the layer-by-layer accumulated thermal stress exceeds the yield strength of the formed material, it will cause local warpage and deformation of the workpiece. Moreover, the large deformation is not conducive to positioning and cutting in the postprocessing process. Therefore, research on efficient, reliable and stable slicing methods and partition scanning strategies is of great significance to reduce part deformation and improve shape and position accuracy.

Currently, in order to improve the efficiency of data processing and molding accuracy, a large number of research studies on Slicing Algorithm and scanning strategy have emerged. Jaiswal et al. [15] sliced the model along the vertical direction before slicing, then identified the thin wall, sharp corner and other features in the model, and finally quantitatively evaluated the formability of different features to find the best forming direction to improve the forming quality. Pandey et al. [16] deduced the recursive formula of slice thickness according to the step effect and the requirements of the surface roughness of the formed part to realize adaptive slicing. Song et al. [17] used the MS algorithm to slice the implicit surface directly, and the efficiency and accuracy of slicing can be controlled by changing the number of sampling points to avoid the accuracy loss caused by the conversion between the hermit surface and STL file. The above algorithm is the mainstream data processing method in the current additive manufacturing process. During the execution of the algorithm, it is necessary to classify the triangular patches or even establish the topological relationship between the patches, which causes a lot of wasted time. For this reason, this article proposes a direct slicing technology of a 3D model based on GPU computing, which performs parallel coloring according to the normal vector and vertex coordinates of the triangular facets in the STL data, and changes the slice position by adjusting the projection matrix to the slice height. After that, the cross-section binary graph is output according to the intersection result of the ray and the triangular facet. Finally, the MS algorithm is used to complete the contour data extraction. The algorithm does not need to sort and delete triangles, and combines the GPU’s concurrency to shorten the contour extraction time.

Manmadhachary et al. [18] used the FFT method to smooth the contour data in the CT image, then used the triangular meshing algorithm to reconstruct and repair the three-dimensional model to improve its surface quality, and finally used the traditional slicing method for slicing. The algorithm has achieved good accuracy in the mandibular model slice test. The above algorithm is mainly suitable for offline processing of the model, and it is difficult to dynamically adjust the process parameters according to the feedback information in the molding process. Jia et al. [19,20] studied the influence of different scanning angles on the deformation of parts under three scanning methods through thermal indirect structural coupling simulation, and found that the 15° rotating scanning strategy produced the least stress, while this scanning strategy had almost no effect on the size of the molten pool. Chun-YuTsai et al. [21] compared the accumulation of residual stress in the part, under the two forming methods of s-shaped scanning and partition scanning, and found that the partition scanning strategy can clearly reduce the tensile stress of the part boundary. The numerical simulation of the optimization of the partitioning method and the scanning strategy mainly focuses on the influence of the angle change of the filling line on the stress and deformation. It is difficult to carry out large-scale calculations in the simulation of the macroscale scanning sequence, so in-depth research needs to be carried out through experimental means.

Due to the large size of the formed part, the traditional STL slicing algorithm needs a long data processing time in the preprocessing stage, which makes it difficult to meet the deformation control requirements of real-time data adjustment. Moreover, the electron beam additive manufacturing is often combined with the numerical control system, so a large number of line data will lead to frequent acceleration and deceleration of the motor in the forming process, resulting in the phenomenon of “tool shaking”. In order to solve the above problems, the research of this article mainly focuses on the following three aspects: (1) The reverse ray tracing algorithm is used to slice the 3D model in parallel, and then the slice position or slice direction is adjusted in real time according to the height feedback information in the forming process. (2) According to the area of the forming layer and the thickness of the part, the number of zones and the scanning sequence of the zones are planned to make the temperature field distribution relatively uniform. (3) The NURBS basis function is used to fit the slice data, combined with the high-order curve processing commands of the CNC system, to reduce the amount of G code transmission while keeping the linear velocity constant.

## 2. Slicing Algorithm Based on Reverse Ray Tracing

**Figure 1:**
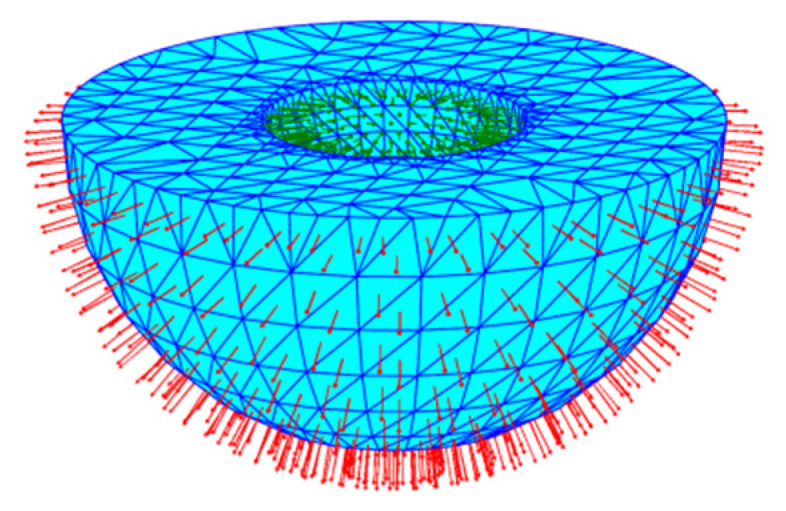
Triangulation strategy of 3D model.

STL (stereolithography) is one of the main data formats used in additive manufacturing, including ascii format and binary format. The generation process of the STL file is to discretize the three-dimensional surface into triangular patches according to the given triangle tolerance, where the three vertices of the outer patch are stored in counterclockwise order, as shown by the red arrow in Figure 1, and the three vertices of the inner patch are stored in counterclockwise order, as shown by the green arrow in Figure 1.

### 2.1. Reverse Ray Tracing Algorithm

The light of the reverse ray tracing algorithm is emitted from the location of the camera, which is different from the object lighting. After that, the light intensity and color value at the intersection of the light and the model are tracked and mapped to the corresponding voxel. The algorithm is mainly used in model rendering and other aspects. In the process of tracking, it needs to iterate along the refraction or reflection direction of the light until the light intensity is less than the set value or projected into the environment. In the 3D model slicing, we only need to consider whether the corresponding voxel is in the model, which has nothing to do with information such as color and texture. Therefore, the refraction and reflection of the light are not considered in the calculation process. When the light intersects the model, the corresponding voxel (pixel) is set to black, otherwise white. In order to ensure the accuracy of contour extraction, the field of view is initialized according to the requirements for contour accuracy during the forming process.

The positional relationship between the STL triangle patch and the space coordinate point x can be expressed by Formula (1).

(1) $$f(x)=\left\{ \begin{array}{l} -1\;\;\;\mathrm{Point}\;\mathrm{outside}\;\mathrm{the}\;\mathrm{model}\\ 0\;\;\;\mathrm{Point}\;\mathrm{on}\;\mathrm{the}\;\mathrm{model}\\ 1\;\;\;\mathrm{Point}\;\mathrm{in}\;\mathrm{the}\;\mathrm{model} \end{array}\right.$$

The ray **r**(*d*) emitted from the camera position is:

(2) r(d)=c+dn   (d>0)

where **n** is the direction vector of the light, and *c* is the camera position coordinate.

When the ray is projected onto the cross-section plane and the projection of the three vertices of the triangular patch satisfies the Formula (3), it means that the ray directed to the pixel point from the viewpoint intersects with the three-dimensional patch. Traverse all the pixels in the field of view in turn to get the binary image of the model at the specified height:

(3) $$\vec{{p_{1}}^{\prime }c\prime }=\alpha \vec{{p_{1}}^{\prime }{p_{2}}^{\prime }}+\beta \vec{{p_{1}}^{\prime }{p_{3}}^{\prime }}\;\;\;(0\leq \alpha +\beta \leq 1)$$

where *p*_1_′, *p*_2_′, *p*_3_′ is the projection of the three vertices of the triangular facet on the cross-sectional plane, and *c*′ is the projection of the light on the cross-sectional plane.

### 2.2. Fast Extraction of the Slice Contour

The electron beam additive manufacturing process is similar to the arc welding technology, but its energy input is higher, and the rapid cooling and heating process in the forming process is more obvious. In order to reduce the stress concentration caused by uneven temperature distribution, current common solutions include using an electron gun to scan and preheat the substrate and partition filling. In the partition filling scheme, the feedback signal for determining whether to partition comes from the model feature recognition. The thin-wall features are filled with parallel lines, and the solid features are filled with partitions and variable angle scan lines. In order to ensure the continuity of the molding process, it is necessary to calculate the filling and scanning methods and slice them before the next layer is processed. The GPU parallel slicing algorithm provides the possibility for a stable and reliable implementation of this method.

According to the visibility judgment result of the triangular patches at each pixel, the template buffer technology is used to render the visible grid as white and the invisible grid as black. It traverses all the pixels in the field of view in turn, and outputs a binary image of the cross-section of the three-dimensional model. Specific steps are as follows:

(1) Load the STL file, obtain the bounding box information (length, width, and height) of the 3D model, initialize the field of view according to the length and width of the model, and enable the write function of the template cache;
(2) Specify the slice height, and fill the template buffer with unsigned integer data 0, which means that the entire field of view does not need to be drawn;
(3) Select the ray emitted from the viewpoint to intersect with all grids in the field of view. When the ray intersects with the forward grid, the value of the template buffer increases by 1, indicating that the pixel needs to be drawn; when the ray intersects with the reverse grid, the value of the stencil buffer decreases by 1, indicating that the pixel does not need to be drawn;
(4) After the traversal is completed, the fragments with the value 1 or 2 in the buffer are rendered, and the fragments with the value 0 are discarded.

**Figure 2:**
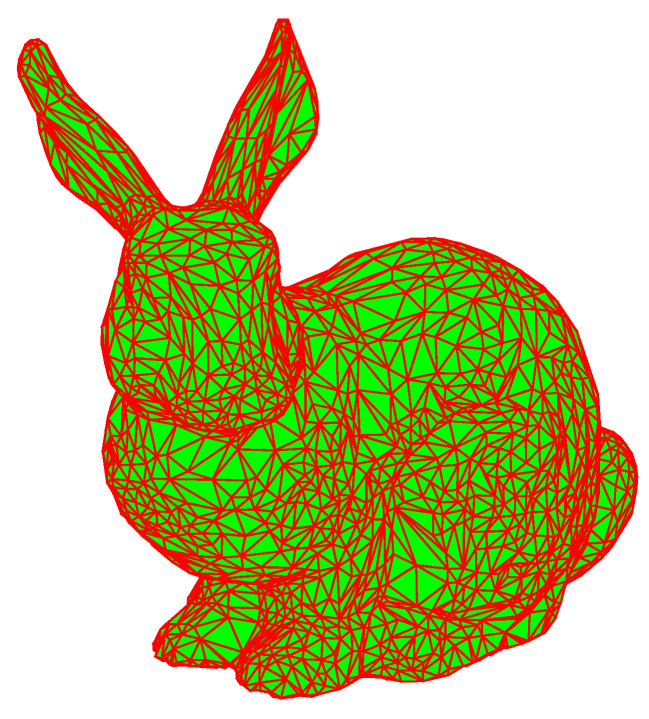
STL triangular patch model.

**Figure 3:**
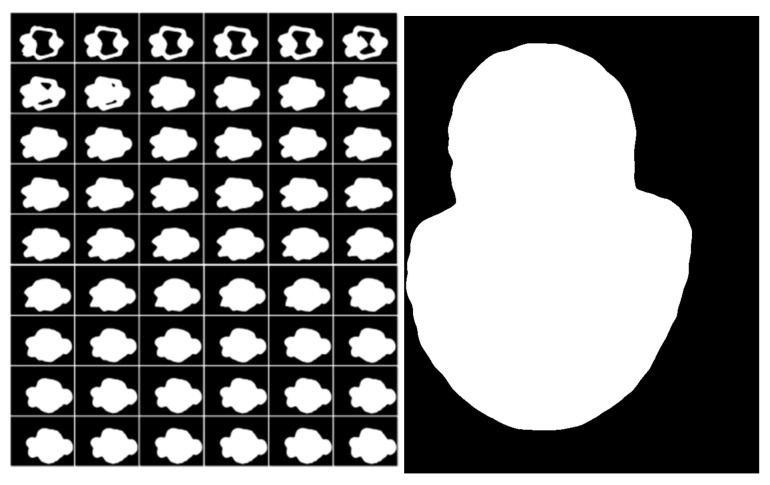
Binary image in frame buffer.

The Stanford bunny shown in Figure 2 is used as the verification model, the layer thickness is set to 0.2 mm, and the pixel sampling accuracy is 0.1 mm. The accuracy can be set according to the requirements of the forming process. By updating the stencil buffer, color buffer and depth buffer data, the model section is finally stored in the frame buffer in the form of a binary image, and the output is shown in Figure 3.

## 3. Contour Extraction and NURBS Fitting Optimization

### 3.1. Marching Square Contour Extraction Algorithm

**Figure 4:**
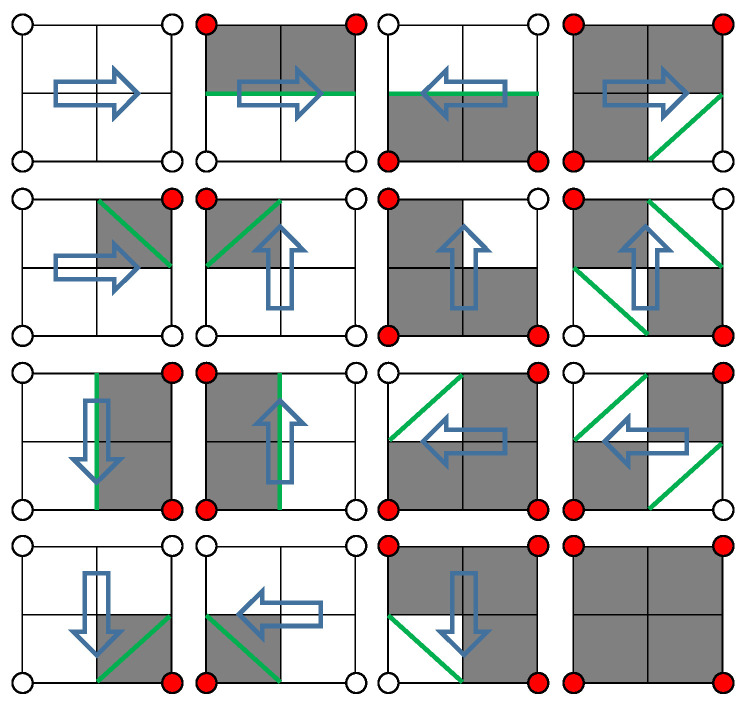
Search direction and slice contour.

Taking the slice shown on the right in Figure 3 as an example, the MS (Marching Square) algorithm is used to calculate all closed contour coordinates. Convert the image data into a two-dimensional grid according to the RGB value of the sampling point coordinates, and mark the color (0, 0, 0) and (255, 255, 255) with the concave and convex state of the grid point. The solid represents the convex grid point, and the hollow represents the concave grid point. Starting from the selected starting point, take out the grid points on the left, top, and top left to form a small block. The states of the four grid points in the block can be divided into 16 situations, as shown in Figure 4. The blue arrow in the figure indicates the direction of the next search. Contour extraction can be completed by sequentially traversing all pixels.

Contour extraction process: (1) Determine the search matrix range according to the position coordinates and color values of the binary image, and initialize the contour matrix to be empty at the same time. (2) Take out 4 pixels, when the colors are all white, search in the order from left to right, from top to bottom. (3) When black appears for the first time among the RGB values of 4 points, mark this point as the search starting point, and store the position coordinates of all black pixels that appear for the first time in the contour matrix. (4) According to the index result of the MS search direction table, move the search range to the next search direction by one-pixel width, as shown in Figure 4. (5) Repeat steps 2–4 until the search process returns to the starting point, which means that the extraction of a closed contour is completed, and then follow the sequence from left to right and top to bottom to find a new search starting point and repeat the above process. (6) Use Formula (4) to interpolate and correct the coordinates of the intersection point.

(4) $$y=y_{0}+\frac{y_{1}-y_{0}}{x_{1}-x_{0}}(x-x_{0})$$

**Figure 5:**
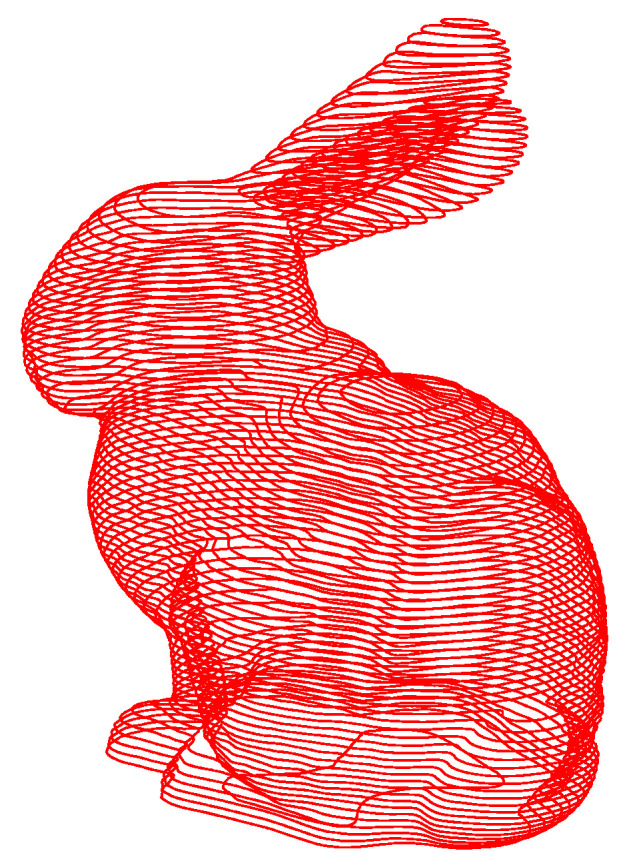
GPU parallel slicing for fast data processing.

The above algorithm is programmed in Python, and the slice data of the Stanford bunny is shown in Figure 5.

### 3.2. Slice Data Fitting Based on the NURBS Basis Function

**Figure 6:**
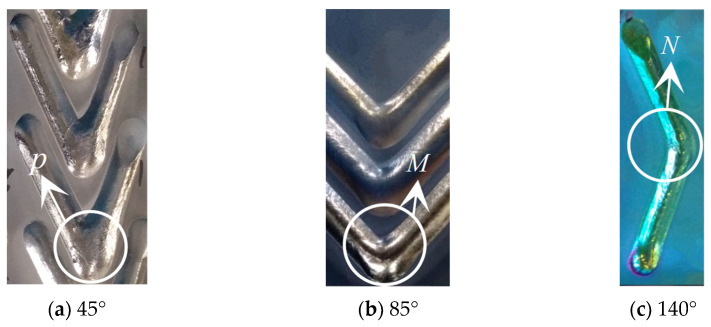
Overaccumulation of metal at the inflection point of small-angle straight-line forming. (**a**) The corner of the polyline is 45°; (**b**) The corner of the polyline is 85°; (**c**) The corner of the polyline is 140°.

The contour generated by STL model slicing is composed of a large number of micro-segment straight lines, and the angle between the small lines is determined by the complexity of the model and the discretization accuracy. During the forming process, the forming speed will be reduced at the inflection point of the line segment to ensure the contour accuracy, but it will increase the quality of the metal fed into the molten pool per unit time, and the formation of metal overaccumulation at the inflection point. This phenomenon becomes more and more serious with the decrease of the included angle, as shown in Figure 6. It is found through experiments that when the straight line included angle is greater than 140°, the over-stacking phenomenon is significantly improved. Therefore, the starting point of a small straight line with an included angle of 140° is used as the dividing point to divide the contour data into a straight-line part and a curved part. After removing the adjacent points, repeated points and internal points of the same straight line, the NURBS curve is used to fit the curve part to reduce the number of starts and stops of the motor to ensure a constant linear speed.

The BSPLINE interpolation function of SINUMERIK 840D SL can smoothly connect discrete coordinate points through control points and weight factors, and use a speed servo to ensure that the linear velocity is constant during the forming process. Suppose the contour coordinate point generated by the slice is {*V_k_*}, *k* = 0, 1, …, *n*, the node vector is U = {*u*_0_, *u*_1_, …, *u*_m_}, the node corresponding to any coordinate point *V_k_* is ${\bar{u}}_{k}$. Therefore, the *n* + 1 linear equations can be constructed based on n coordinate points as shown in (5).

(5) $$V_{k}=S({\bar{u}}_{k})=\sum_{i=0}^{n}N_{i,p}({\bar{u}}_{k})C_{i}$$

where the control point **C***_i_* is the quantity to be determined, and the value range of the node value ${\bar{u}}_{k}$ belongs to [0, 1].

Additive manufacturing is mainly used to manufacture parts that cannot be processed or are difficult to process by conventional methods. Most of its models contain complex curves and surfaces, resulting in a high proportion of C^0^ continuous sharp points or large curvature transition data points in the slice data. Therefore, the coordinate points are classified before fitting, and then the centripetal parameterization method is used to parameterize the curve [22].

(6) $${\bar{u}}_{k}={\bar{u}}_{k-1}+\frac{\sqrt{\left|V_{k}-V_{k-1}\right|}}{\sum_{k=1}^{n}\sqrt{\left|V_{k}-V_{k-1}\right|}},\;\;k=1,2,\cdots ,n-1$$

where ${\bar{u}}_{0}$ = 0, ${\bar{u}}_{n}$ = 1.

To ensure that the tangents of the curve on both sides of the dividing point are collinear, the first-order derivative vectors at the endpoints are set to *F*_0_ and *F_n_*, respectively. The third-order spline curve is used for coordinate point fitting, and the repetition of the curve at the end point is three.

(7) $$u_{j+d}=\bar{u_{j}},j=1,2,\cdots ,n-p$$

where ${\bar{u}}_{0}$ = … = ${\bar{u}}_{3}$ *=* 0, ${\bar{u}}_{n+3}$ = … = ${\bar{u}}_{n+6}$ *=* 1.

Under the above conditions, the equation of the end point of the curve and the first-order derivative equation controlling the direction of the curve are as shown in (8).

(8) $$\left\{ \begin{array}{l} C_{0}=V_{0}\\ -C_{0}+C_{1}=\frac{u_{4}}{3}F_{0}\\ -C_{n+1}+C_{n+2}=\frac{1-u_{n+2}}{3}F_{n}\\ C_{n+2}=V_{n} \end{array}\right.$$

Except for the endpoints, the repeatability of each internal node is 1. From the nature of the cubic spline curve, it can be seen that there are at most 3 non-zero basis functions for the node *u_j_*, so the *n* − 1 equations excluding the endpoints can be expressed by (9)

(9) $$V_{k}=N_{k,3}({\bar{u}}_{k})C_{k}+N_{k+1,3}({\bar{u}}_{k})C_{k+1}+N_{k+2,3}({\bar{u}}_{k})C_{k+2}$$

where, *k* = 1, 2, …, *n −* 1, *a_k_ = N_k,_*_3_(${\bar{u}}_{k}$), *a_k_ = N_k,_*_3_(${\bar{u}}_{k}$), *a_k_ = N_k,_*_3_(${\bar{u}}_{k}$). The linear equations with a coefficient of 2 can be solved by (1–3).

(10) $$\left[\begin{array}{c} V_{1}-a_{1}C_{1}\\ V_{2}\\ \vdots \\ V_{n-2}\\ V_{n-1}-c_{n-1}C_{n+1} \end{array}\right]=\left[\begin{array}{l} b_{1}\;\;c_{1}\;0\;\;\cdots \;0\;\;0\;\;0\\ a_{2}\;b_{2}\;c_{2}\;\cdots \;0\;\;0\;\;0\\ \vdots \;\;\;\;\vdots \;\;\;\;\vdots \;\;\;\;\;\;\vdots \;\;\;\;\;\;\;\vdots \;\;\;\;\;\;\;\vdots \\ 0\;\;\;0\;\;\;0\cdots a_{n-2}\;\;\;b_{n-2}\;\;\;\;\;c_{n-2}\\ 0\;\;\;0\;\;\;0\;\cdots \;\;0\;\;\;\;\;\;\;\;a_{n-1}\;\;\;b_{n-1} \end{array}\right]\left[\begin{array}{c} C_{2}\\ C_{3}\\ \vdots \\ C_{n-1}\\ C_{n} \end{array}\right]$$

**Figure 7:**
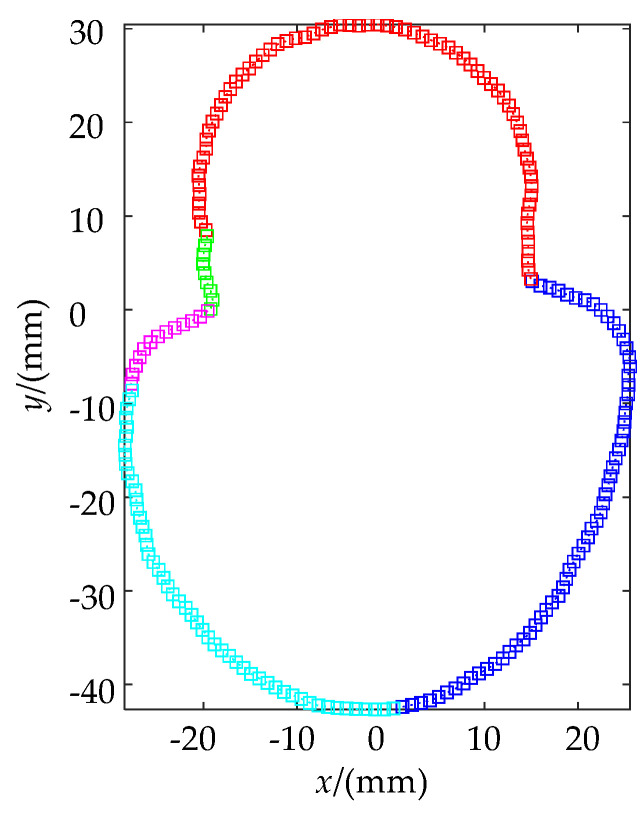
NURBS fitting effect of slice data.

There are no continuous sharp points and large curvature transition segments of C^0^ in the slice data of the 118th layer. The data is divided into 5 curve parts with a critical angle of 140°, and the fitting effect is shown in Figure 7.

## 4. Contour Partition and Scan Filling Strategy

In the forming process of large parts, the temperature gradient is particularly obvious at both ends of the large scan line [23]. The EBAM forming part is a compact blank of the final part and often requires subsequent finishing processing. Excessive deformation may cause the entire part to be scrapped. Therefore, the traditional linear scanning method is difficult to apply to this forming process. The research of Lee et al. [24,25] shows that the temperature gradient and stress concentration can be reduced by partitioning and scanning sequence planning, thereby controlling the deformation of the part within a reasonable range.

### 4.1. The Smallest Enclosing Rectangle of Contour Data

Initializing the position of the filling pattern according to the short side length of the minimum circumscribed rectangle of the slice contour can greatly reduce the number of calculations. Suppose the initial envelope of a certain contour data is {[*x*_0_min_, *y*_0_min_], [*x*_0_max_, *y*_0_max_]}, then the minimum value of Equation (11) is the minimum envelope rectangle required.

(11) $$\begin{array}{l} f=(x_{i\_\max}-x_{i\_\min})(y_{i\_\max}-y_{i\_\min})\\ i=0,\pi /10,2\pi /10,\cdots ,\pi \end{array}$$

A hexagon with side length *d* is used as the pattern to be filled, and the short side and long side of the envelope rectangle are respectively *L*_1_ and *L*_2_. If round(*L*/*d*) + 1 is an odd number, then *L*/*d* + 1 hexagons will be uniformly distributed from the centroid point of *L*_1_ at a distance 1.732*d*; if round(*L*/*d*) + 1 is an even number, then *L*/*d* + 2 hexagons will be uniformly distributed from the centroid point of *L*_1_ at a distance 1.732*d*; The centroid point coordinates of the hexagon in the *L*_2_ direction are updated according to the plane mosaic rule of the polygon.

### 4.2. Partition Filling and Scanning Strategy

**Figure 8:**
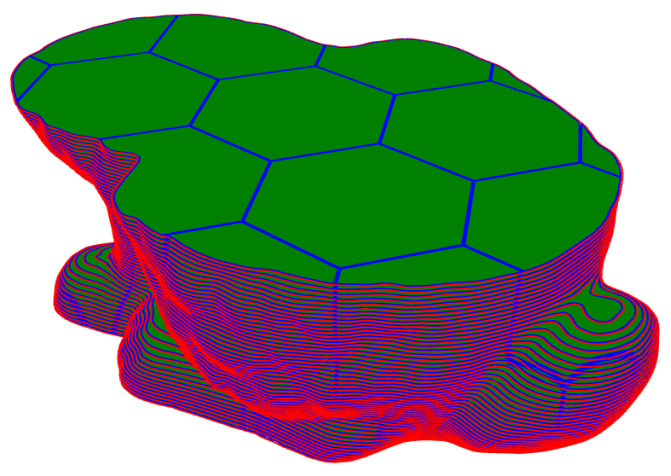
Hexagonal partition and scan filling of slice data.

The offset and intersection operation between the hexagon and the contour curve is realized by the polygon clipping algorithm [26]. Before forming, randomly select a hexagonal area represented by a centroid point as the first forming area, and read the coordinates of the centroid point from the memory to ensure that the Euclidean distance between this coordinate and all processed centroids is the smallest. According to this algorithm, the hexagonal partition of the Stanford bunny is shown in Figure 8.

## 5. Discussion

### 5.1. Slicing Efficiency

**Figure 9:**
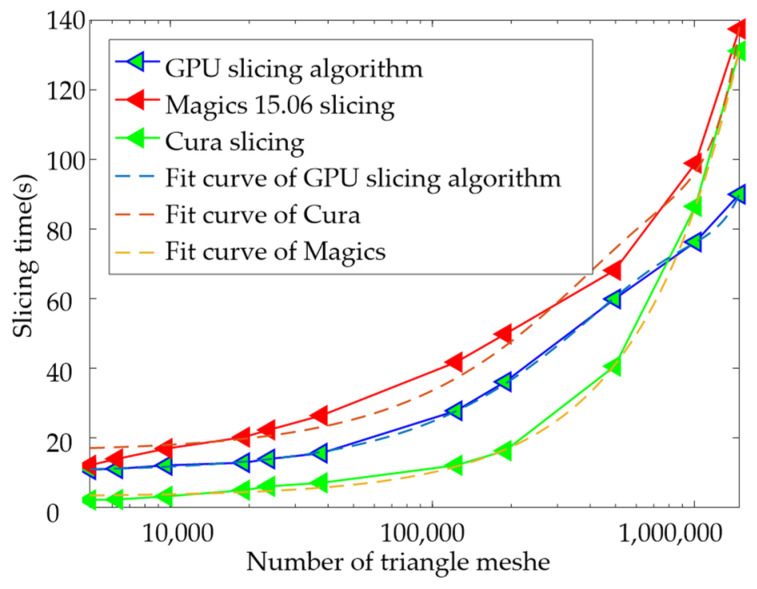
Hexagonal partition and scan filling of slice data.

Use python as the slicing kernel to load vertex data in parallel. The computer hardware configuration is as follows: the graphics card is Intel(R)UHD Graphics 630, and the GPU memory is 128 Mb. Use δ as the triangular tolerance to output 11 different sizes of binary STL models Q, and the slicing efficiency is shown in Figure 9. Where δ(mm) ∈ [1, 0.4, 0.8, 0.08, 0.06, 0.04, 0.01, 0.006, 0.004, 0.002, 0.001], Q(MB) ∈ [0.23, 0.30, 0.46, 0.91, 1.14, 1.80, 5.88, 9.04, 23.65, 48.05, 70.73].

The efficiency of the GPU slicing algorithm during the entire test was significantly higher than that of Cura. The slicing efficiency of commercial software Magics is the highest, and the CPU utilization rate of the stable slicing process is kept at 29.45%, but the process will be stuck during the operation. As the number of triangular patches increases, the time to establish the topological relationship between Cura and Magics will gradually increase, which is one of the reasons why the slicing time changes approximately exponentially. The GPU slicing algorithm does not need to establish a topological relationship and only needs to traverse the voxels at the specified height. As the number of grids increases, the parallel computing causes the GPU utilization to gradually increase, up to 93%, so the last model slicing only takes 89.99 s.

### 5.2. Analysis of Curve Fitting Error

**Figure 10:**
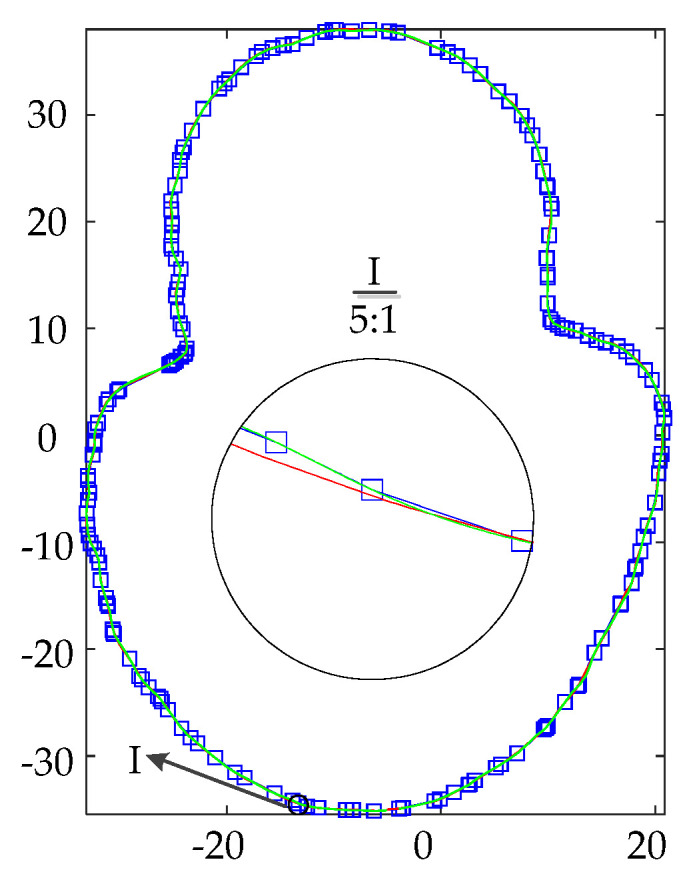
Slicing data and contour fitting.

The linear and spline basis functions are used to fit the contour coordinates. The fitting effect is shown in Figure 10. The blue curve is the slice contour of the high-precision model, and it is used here instead of the original contour. The red curve is the slice contour of the medium precision model. The NURBS curve can approach the blue contour line to restore the original data according to the extension trend of the original data.

**Figure 11:**
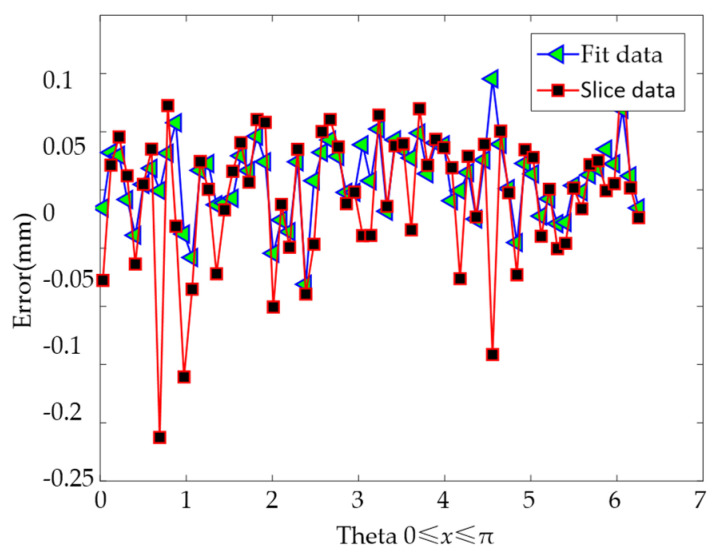
Approximation error of sliced data and fitted data.

Taking the centroid of the enveloping rectangle of the contour data as the extreme point, the polar diameter changes of the three curves are calculated to represent the error distribution of the fitting data and the slice data. The results are shown in Figure 11. The error range between the slice data of the medium-precision model and the slice data of the high-precision model is (−0.2125, 0.1137) mm, and the error range of the fitted data and the slice data of the high-precision model is (−0.0810, 0.0955) mm. Therefore, the error of the contour data is reduced by 34.2% after fitting.

## 6. Conclusions

Aiming at the problem of excessive metal accumulation at the inflection point in the forming of large parts, NURBS basis functions are used to fit the contour data to ensure a constant forming line speed. The results show that when the triangle tolerance is one, the segmental fitting of the slice data through the spline basis function can reduce the conversion error of the STL file by 34.2%, and at the same time reduce the over-stacking phenomenon of the inflection point of the straight line. Aiming at the problem of warpage and deformation caused by too long scanning line length, a partition scanning strategy is proposed. Planning the scan sequence according to the centroid distance of the partition pattern can significantly reduce the length of the scan line in the model, and increase the molding time interval of adjacent regions at the same time. Aiming at the dynamic adjustment of the layer thickness of large parts during the forming process, a slicing algorithm based on the reverse ray tracing algorithm is proposed. This algorithm has a high slicing efficiency and can calculate slices and partition filling data in the printing gap. By comparing it with other similar algorithms, it is found that when there are a large number of triangular patches, using the GPU slicing algorithm to load voxel information in parallel can significantly reduce the calculation time. The research results in this paper can provide new ideas for the dynamic adjustment of layer thickness and deformation control in the forming process of large parts.

## Data Availability

The data presented in this study are available on request from the corresponding author.
